# Graphitic Carbon Electrodes on Flexible Substrate for Neural Applications Entirely Fabricated Using Infrared Nanosecond Laser Technology

**DOI:** 10.1038/s41598-018-33083-w

**Published:** 2018-10-03

**Authors:** Maria Vomero, Ana Oliveira, Danesh Ashouri, Max Eickenscheidt, Thomas Stieglitz

**Affiliations:** 1 Laboratory for Biomedical Microtechnology, Institute of Microsystem Technology (IMTEK), University of Freiburg, Georges-Koehler-Allee 102, D-79110 Freiburg, Germany; 2 Cluster of Excellence BrainLinks-BrainTools, University of Freiburg, Georges-Koehler-Allee 80, 79110 Freiburg, Germany; 3 Bernstein Center Freiburg, University of Freiburg, Hansastrasse 9a, 79104 Freiburg, Germany

## Abstract

Neural interfaces for neuroscientific research are nowadays mainly manufactured using standard microsystems engineering technologies which are incompatible with the integration of carbon as electrode material. In this work, we investigate a new method to fabricate graphitic carbon electrode arrays on flexible substrates. The devices were manufactured using infrared nanosecond laser technology for both patterning all components and carbonizing the electrode sites. Two laser pulse repetition frequencies were used for carbonization with the aim of finding the optimum. Prototypes of the devices were evaluated *in vitro* in 30 mM hydrogen peroxide to mimic the post-surgery oxidative environment. The electrodes were subjected to 10 million biphasic pulses (39.5 μC/cm^2^) to measure their stability under electrical stress. Their biosensing capabilities were evaluated in different concentrations of dopamine in phosphate buffered saline solution. Raman spectroscopy and x-ray photoelectron spectroscopy analysis show that the atomic percentage of graphitic carbon in the manufactured electrodes reaches the remarkable value of 75%. Results prove that the infrared nanosecond laser yields activated graphite electrodes that are conductive, non-cytotoxic and electrochemically inert. Their comprehensive assessment indicates that our laser-induced carbon electrodes are suitable for future transfer into *in vivo* studies, including neural recordings, stimulation and neurotransmitters detection.

## Introduction

In the field of neural prostheses, much attention is now given to the long-term performance of the materials directly interfacing with the nervous system^1–6^. Neural interfaces indeed play a critical role in chronic applications, where they have to outlast the highly humid and oxidative body environment without undergoing delamination or corrosion and thus losing their functionality over time^7–13^. Among all, carbon was proved to be the material with the highest potential to simultaneously serve as biomaterial for recording nerve cells activity, electrically stimulating them and, in addition, for selectively detecting the presence of neurotransmitters and other electrically active biomolecules^14–18^. However, the batch fabrication of carbon electrode arrays and their integration into micromachining technologies for flexible substrates represent key challenges that often limit the usage and the investigation of carbon as electrode material for neural interfaces. The feasibility of the fabrication method - in terms of process complexity and cost - is a factor of great importance and it is not always easy to accomplish with the current carbon technology. In fact, the high temperature (>900 °C) and long process time (6 to 12 hours) required for conventional pyrolysis could be limiting factors for the manufacturing of carbon electrode arrays. Such process parameters also highly complicate the stable integration of carbon into polymer-based micromachined devices. Additionally, the metal components of micromachined electrode arrays are usually very thin (in the range of nanometers) and challenge mechanical and electrochemical stability^19–21^. For these reasons, laser technology was recently investigated and developed as an alternative for mid-scale integration densities to traditional photolithographic methods, not only for patterning the silicone rubber substrate and platinum/iridium electrodes and tracks^22–24^, but also for carbonizing the electrode sites^25^.

In our recent study, we have demonstrated that with laser technology it is actually possible to rapidly and locally carbonize carbon-based polymers, such as parylene C, when they are used as coatings for metal components (e.g. platinum/iridium tracks)^25^. With this method, we have therefore fabricated microelectrode arrays with laser-induced carbon electrode sites and platinum/iridium tracks on a flexible silicone rubber and parylene C substrate. In such devices, laser technology interestingly substitutes both conventional microfabrication processes - for thin and thick film manufacturing - and pyrolysis processes - for the production of graphitic carbon components. The referred fabrication method surely has the potential to have a high impact on the carbon electrodes technology towards clinical trials, as it represents a rapid, simple and economic way to make on-demand electrodes for patients with different anatomies and for various applications where carbon can improve the performance of the implanted devices (e.g. cuff electrodes for peripheral nerve stimulation, retinal implants, ECoG and micro-ECoG arrays for brain-computer interfaces (BCI) and deep brain stimulation (DBS) devices for closed-loop systems). Such devices have electrode-site dimensions that are rather suited to record local field potentials and mass signals or stimulate populations of fibers and cell bodies, than for single unit access. In the current study, we investigated and optimized this laser technology by using two selected laser pulse repetition frequencies (20 kHz and 40 kHz) to carbonize the electrodes with the aim of identifying the most suitable one for clinical neural applications. With this in mind, we performed elemental analysis and a series of *in vitro* and *in situ* experiments, including electrical and electrochemical aging and cell viability tests. Our goal was to find a rapid way for prototyping robust graphitic carbon electrode arrays on flexible substrates, optimize their performance, and potentially refine the role of carbon as electrode material in the field of neural prostheses.

## Results and Discussion

**Figure 1:**
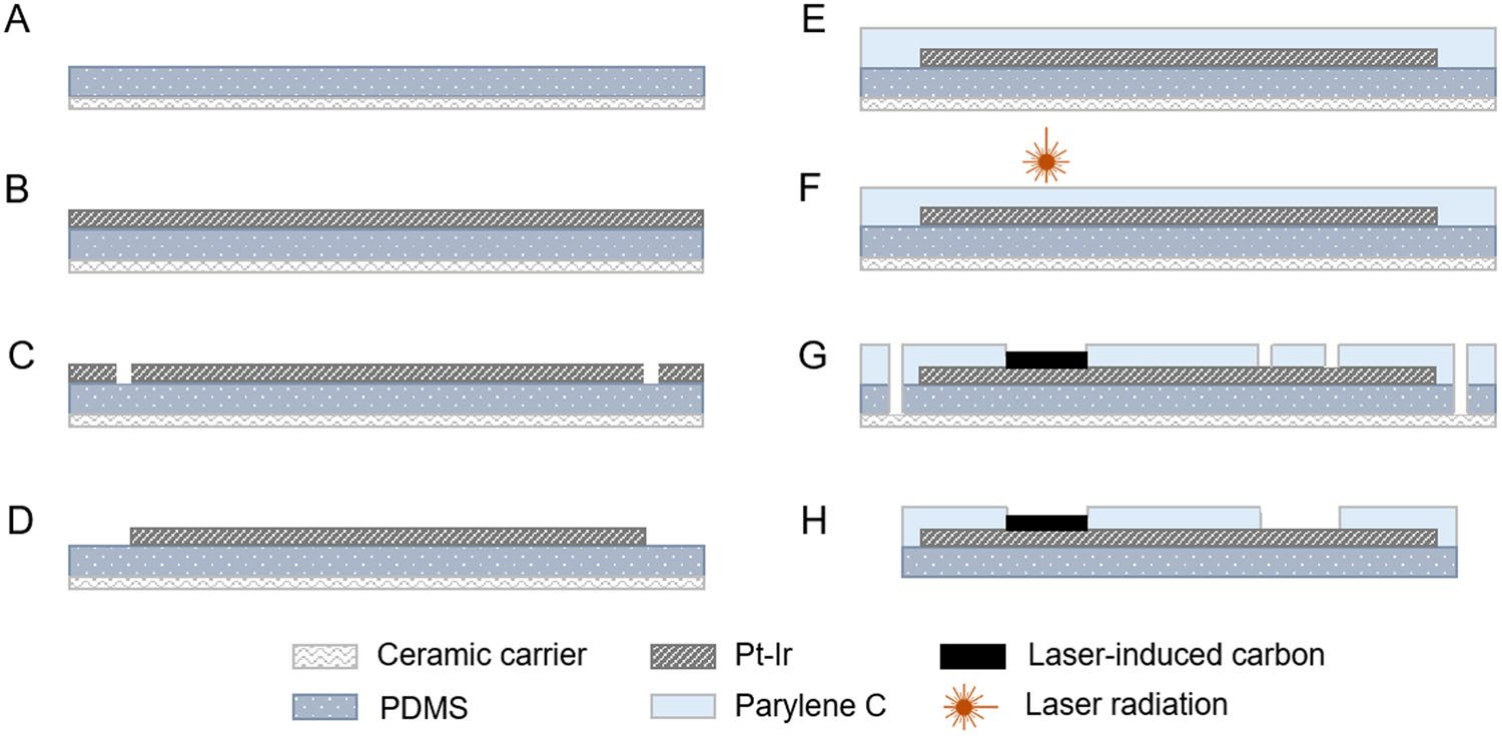
Schematic of the fabrication process of the electrode arrays. (**A**) Spin-coating of silicone rubber onto a ceramic carrier. (**B**) Lamination of the metal layer. (**C**) Structuring of the metal. (**D**) Removal of the metal excess. (**E**) Parylene C coating. (**F**) Laser pyrolysis of the active site and structuring of the electrodes (700 µm in diameter). (**G**) Opening of the metal pads. (**H**) Device releasing.

The ECoG (electrocorticography) devices studied here were manufactured using laser technology as one and only manufacturing tool. The basics of the fabrication method for manufacturing laser-induced carbon electrode arrays are explained in details elsewhere^25^ but the main fabrication steps are shown in Fig. 1. In synthesis, a 200 µm layer of medical grade silicone rubber was spun onto a ceramic carrier and a platinum/iridium foil (25 µm) was laminated onto it before the complete curing of the silicone. The metal foil was patterned in the shape of electrodes and tracks (140 µm wide), and parylene C (10 µm) was then deposited on top as insulation for the metallic components. Afterwards, the electrode sites were laser-structured in an inert atmosphere (N_2_) to obtain laser-induced carbon electrodes and finally the devices were released from the ceramic carrier.

**Figure 2:**
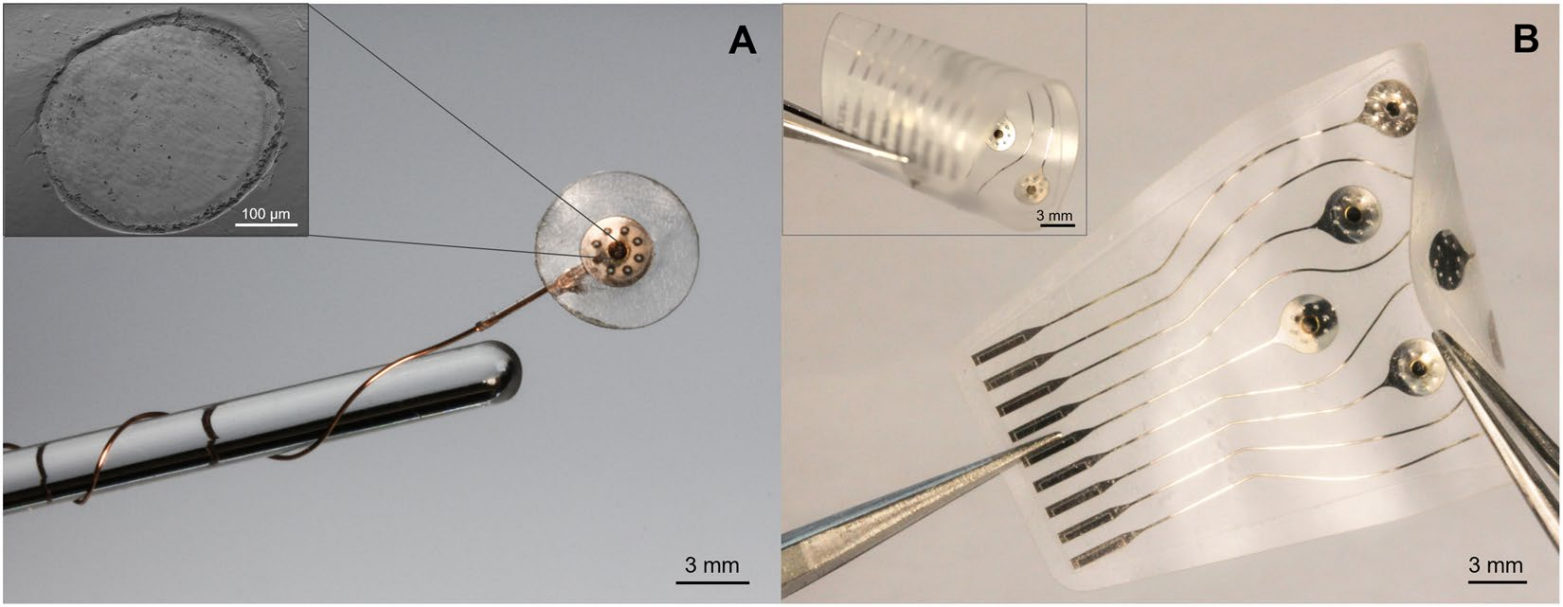
Pictures of (**A**) a single-channel device - with SEM (scanning electron microscopy) picture of the carbon electrode in the inset - and of (**B**) an ultra-flexible 9-electrode array (curled in the inset). Both were manufactured with the method described above.

This fabrication process led to the realization of flexible micro-ECoG arrays of various shapes and dimensions. Two examples are shown in Fig. 2, where a single-channel device is pictured on the left (Fig. 2A) and a 9-electrode array on the right (Fig. 2B). For simplicity, single-electrode devices - similar to the one shown in Fig. 2A - were used for the extensive *in vitro* characterization of the laser-induced carbon material. Upscaling of electrodes number and density, as well as miniaturization of tracks and electrodes, can be easily performed by changes in the design file used as source for the laser writing step^6,26^. The resolution of the features obtained by laser structuring the devices is a function of laser’s beam focus (about 30 µm in case of the infrared nanosecond laser used here) and the processed material. It was demonstrated, for instance, that the nanosecond pulse regime allows the fabrication of 25 µm-wide microelectrode tracks, with a clearance of 60 µm^27^. When used for carbonization in a ‘line-writing’ manner though – consisting in writing lines to ‘fill in’ a certain geometry (in our case disk-shaped electrodes) – it allows the carbonization of electrodes with a diameter of 200 µm or larger.

We experimentally found that the laser parameters with more influence on the carbonization process are laser power and pulse frequency and that, for obtaining the most conductive carbon and cleanest features, the best combination of these two parameters is a power of 1.6 W and a frequency range between 20 and 40 kHz. Other combinations either led to overly burned polymer (power >40%) or little to no carbonization (power <40%) (see Fig. S1 in supplementary information). This parametric approach allowed to find the compromise between minimum energy required for initiating the carbonization process of parylene C and maximum heat tolerated by the underlying metal tracks before starting deforming. Laser-induced carbon was, in fact, obtained by heating up the metallic substrate (platinum/iridium) through an infrared laser beam and, doing so, transferring the heat to the parylene C insulation that carbonized and became conductive. Direct heating of the semi-transparent parylene C by the laser beam is negligible due to the polymer’s low absorption coefficient^28^ and its large penetration depth for infrared light. On the other hand, laser absorption on the platinum surface is sufficient to cause a strong temperature increase in the space-time expansion of the laser pulse. Between pulses, the surface cools down again within a few microseconds after the pulse^29^, whereby the heat spreads into the bulk and the interfacing layer but does not affect the integrity of the silicone rubber underneath. A certain amount of heat is transferred to the parylene C and conserved there, as the parylene’s thermal diffusivity (D = k/(c) = 0.1 µm^2^/µs) is very small compared to the laser’s spot diameter (30 µm) and its pulse frequency range (10–100 kHz). Here, since ρ = 1.29 g/m³ is the mass density, c = 0.71 J/(Kg) is the specific energy and k = 0.084 W/Km the thermal conductivity (information provided by the supplier Speciality Coating Systems-SCS), it can be assumed that when the laser pulse repetition doubles, the temperature at the parylene C interface doubles as well. Because the temperature is a crucial factor during every carbonization process^14^, the laser parameters should be modulated and optimized to yield crystalline graphitic structures. To achieve that and have a better understanding of the influence that the laser pulse frequency has on the structural and behavioral changes of our carbon electrodes, we then directly compared two groups of devices: the P20 group, made by using an infrared nanosecond pulsed laser with a working frequency of 20 kHz, and the P40 group, made using the same laser but with a working frequency of 40 kHz. The two groups of devices were manufactured using identical fabrication steps, they differed only in the laser parameters used for the laser-induced carbonization of the active sites. The aim of this study was to determine initial structural and behavioral differences of the laser-induced carbon electrodes, as well as their short and long term performance. Therefore, several *in vitro* tests were performed to investigate these aspects.

**Figure 3:**
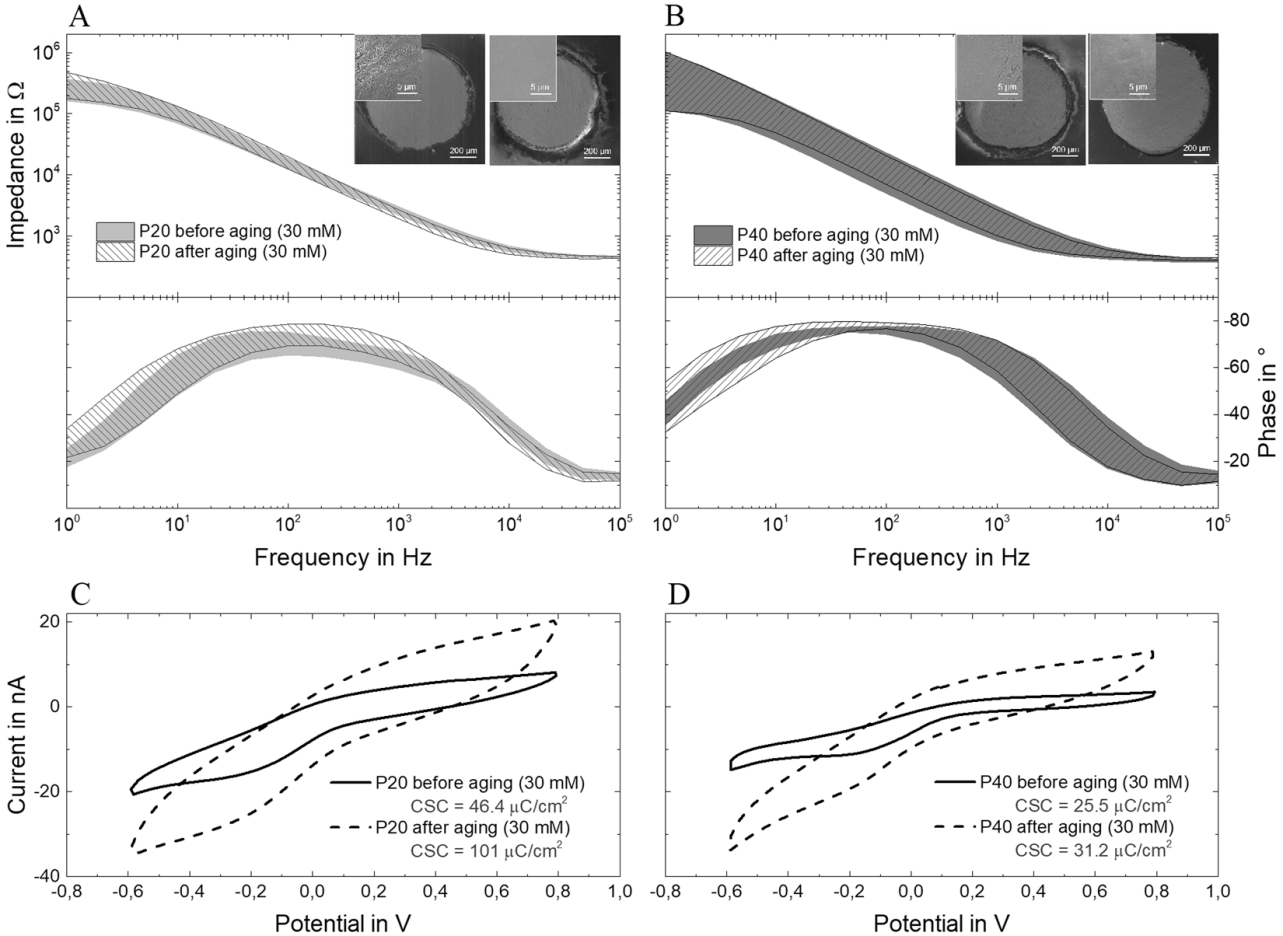
EIS measurements (magnitude and phase) of four electrodes per group, P20 (**A**) and P40 (**B**), before and after immersion in 30 mM H_2_O_2_ for one week at 37 °C_._ Shade regions consider the average and standard deviation of the obtained data points. Cyclic voltammograms of a (**C**) P20 and (**D**) P40 electrode (**C**) before and after immersion in 30 mM H_2_O_2_ for one week at 37 °C.

To evaluate the durability of the electrodes in chronic implantations, four devices of each group - P20 and P40 - were aged in a solution of phosphate buffered saline (PBS) and 30 mM hydrogen peroxide (H_2_O_2_). The devices were left in solution for one week, at constant temperature of 37 °C and protected from UV light. The presence of H_2_O_2_ is intended to simulate the *in vivo* environment surrounding the electrodes during the acute post-surgery tissue response to the implant, where reactive oxygen species (ROS) are released by the brain immune cells to attack the foreign body for the first 7 days after the implantation^30^. After one week, electrochemical impedance spectroscopy (EIS) measurements were taken to determine whether the devices remained functional. Figure 3 shows the average and standard deviation of the EIS measurements data (Bode plot with magnitude and phase) of the laser-induced carbon electrodes before and after the aging, for both groups. It additionally shows the scanning electron microscope (SEM) images of the electrodes before and after the treatments (insets) and the voltammograms of a P20-type and a P40-type electrode before and after immersion in 30 mM H_2_O_2_ (Fig. 3C,D, respectively).

Results demonstrate that the two groups (P20 and P40) have a very similar electrochemical behavior, although P40 appears to be the group with higher variability among electrodes of the same kind. The electrochemical behavior of the two group types does not change significantly after the aging in 30 mM H_2_O_2_, in particular the moduli of both groups look nearly superimposable to the ones measured before the aging test. EIS plots of individual electrodes are reported in the supplementary information (Fig. S2). It should be noted that the phase of the aged electrodes shifts and becomes more capacitive at the low frequencies both for P20 and P40, although it maintains the same shape. The same change happens to the platinum iridium control case (see Fig. S3 in supplementary information). The cyclic voltammograms show an increase in area and thus in charge storage capacity for both electrode types, with no additional cathodic or anodic peaks, and an increase in current. SEM images confirm that the devices did not delaminate nor crack during the test. Overall, there are no significant changes in the behavior of the laser-induced carbon electrodes due to the performed aging test, meaning that the devices have the potential to withstand the post-surgery corrosive environment.

**Figure 4:**
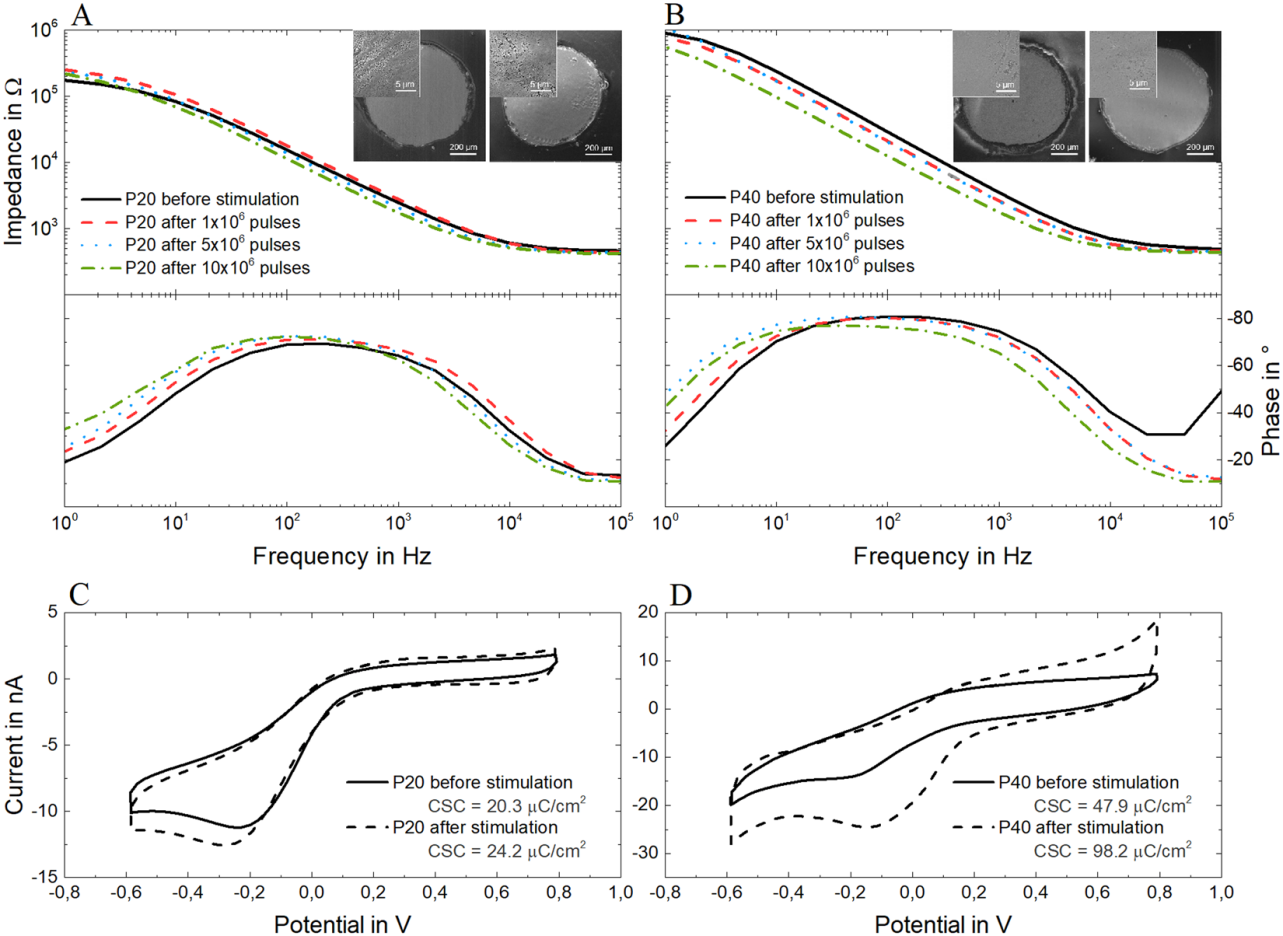
EIS measurements (magnitude and phase) of a representative electrode of each group - P20 (**A**) and P40 (**B**) - before the electrical stimulation test and after 1, 5 and 10 million biphasic pulses. Cyclic voltammograms of electrodes P20 (**C**) and P40 (**D**), before the electrical stimulation test and after 1, 5 and 10 million biphasic pulses.

**Figure 5:**
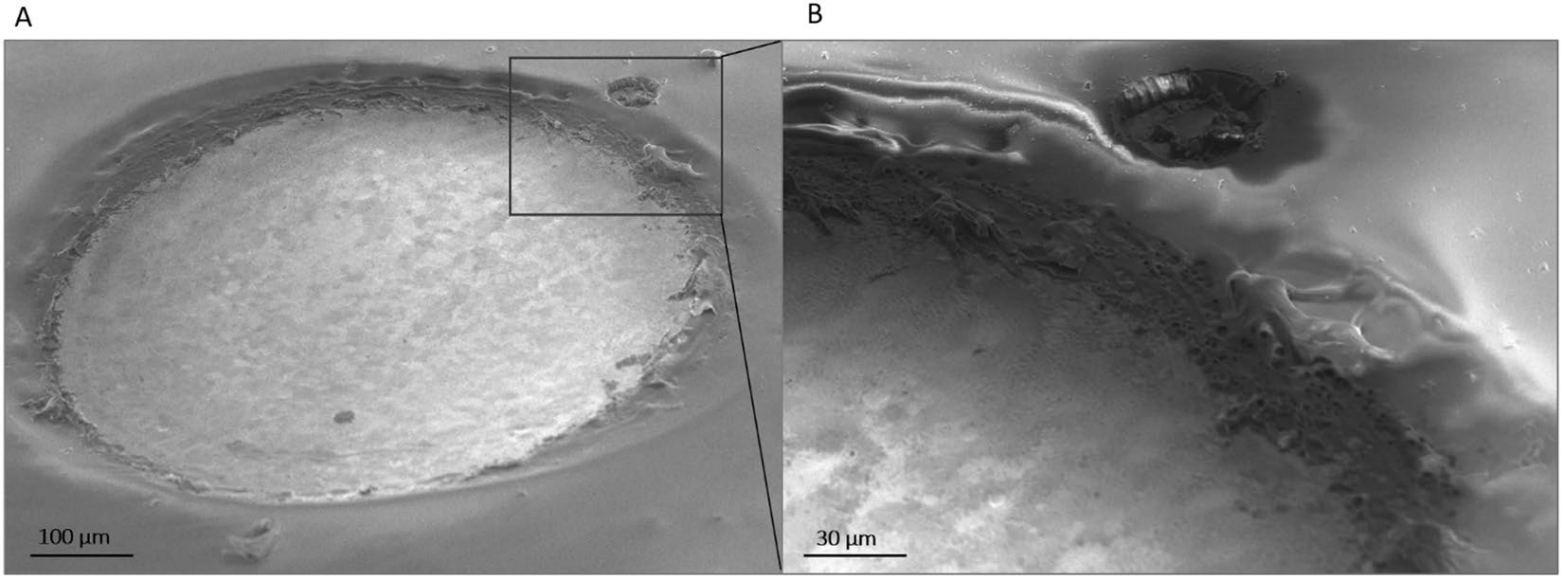
SEM images of a P40-type laser-induced carbon electrode after electrical stimulation (10 million biphasic current pulses). No delamination is observed in the overall picture (**A**) nor in the detail (**B**) taken at 1000x magnification.

A separate batch of laser-induced carbon electrodes was then stimulated with charge balanced biphasic current pulses, to test their ability to resist electrical stimulation. As for the aging tests, two groups of devices - P20 and P40 - were subjected to the same protocols: prolonged stimulation patterns were applied and EIS measurements were performed at intermediate stages (before stimulation and after 1, 5 and 10 million pulses) to monitor the devices’ behavior and identify eventual changes. In total, 10 million pulses were applied to each electrode. The water window of the laser-carbon electrodes was measured - by means of linear sweep voltammetry - to avert the possibility of triggering irreversible reactions during stimulation and it was estimated to be about 2.7 V wide (from −1.0 V to 1.7 V, see Fig. S4). The voltage response of the electrodes was monitored during the stimulation to ensure that the safe voltage window was never approached or exceeded. Figure 4 shows the EIS measurements of a representative case per group, performed before starting the stimulation test and then after 1, 5 and 10 million biphasic pulses together with the corresponding SEM images of the electrodes before and after the test. The behavior of P20 and P40 is similar during the test, with the magnitude remaining almost identical to the one measured before the stimulation and the phase shifting at the low frequencies and becoming more capacitive, in both cases. The low frequency phase shift seems proportional to the number of pulses applied to the electrodes. For most of the P40-type electrodes (see Fig. 4B, for example), the magnitude of the impedance decreases proportionally to the number of pulses applied. A similar behavior is reported in literature for other kinds of pyrolyzed carbon electrodes (e.g., glass-like or glassy carbon) and it is interpreted as a sign of their activation^9,10^. The electrochemical behavior of both groups of electrodes subjected to electrical stimulation is interestingly comparable to the behavior seen during the aging test, with the exception of the magnitude of the P40 electrodes. This suggests that laser-induced carbon, when made with a laser frequency of at least 40 kHz, possibly undergoes an activation phase - when electrically polarized - during which its impedance and surface roughness change^10,31^. The cyclic voltammograms of P20 and P40 electrodes (Fig. 4C,D) confirm an increase in charge storage capacity for both electrode types, which is though much more noticeable for the P40 type. The SEM images in the insets of Fig. 4 confirm that both the electrode types remained intact and did not delaminate. Furthermore, their macroscopic morphological characteristics do not seem to change after biphasic pulse stimulation and the interface with the surrounding parylene C looks intact even in the P40-type, which is the carbon type that changed the most after stimulation (Fig. 5).

An equivalent electrical circuit model was used to interpret the experimental EIS data and the charge transfer process happening on the electrodes surface, as well as the behavior of the laser-induced carbon electrodes after the aging and stimulation experiments. The most representative equivalent circuit for these electrodes is the Randles cell model with access resistance (R_S_), constant phase element impedance (Z_CPE_), charge transfer resistance (R_ct_) and Warburg diffusion impedance (Z_W_), in the configuration shown in the schematic of Table 1. The electrodes’ access resistance, mainly dependent on their geometry and on the electrolyte, seems to be unaffected by the process parameters or treatments. Therefore, delamination or enlargement of the electrodes areas (due to removal of fabrication residues, peeling of the carbon particles, or electrochemical dissolution with surface roughening) can be excluded. This assumption is corroborated by the post-treatment optical characterization (Fig. 5). The same is true for the constant phase element (CPE):

1 $${Z}_{CPE}=\frac{1}{T\cdot {(i\omega )}^{n}}$$

whose impedance, given by the variables T and n, does not significantly vary before and after the treatments. Here, *i* is the imaginary number and ω is the angular frequency. The constant phase element impedance (Z_CPE_) correlates to the energy dispersion at the electrode-electrolyte interface and can be affected by a series of factors, such as micro-fractal surface roughness^32,33^ or non-uniform current distribution^34^.

**Table 1:** EIS parameters of electrodes P20 and P40, before and after aging and stimulation, obtained by fitting the experimental data to the model shown.

		χ^2^/%	R_S_/Ω	T_0_/nS∙s^−n^	n	R_CT_/Ω	T_0_/kS∙s^−0.5^	C/nF
P20	pristine	0.32	426	467	0.79	119	33	31
	Aged	0.24	426	405	0.82	143	37	44
P40	pristine	0.74	405	272	0.88	647	29	70
	Aged	0.69	424	236	0.89	437	133	71
P20	pristine	0.40	499	147	0.89	584	91	42
	stimulated	1.31	428	185	0.90	1285	82	64
P40	pristine	0.89	428	648	0.85	206	83	122
	stimulated	0.56	413	435	0.89	539	24	139

In addition, the goodness of the fit parameter (χ^2^) and the approximated capacitance C are listed.

Additionally, the Warburg impedance, defined as:

2 $${Z}_{W}=\frac{1}{{Y}_{W}.\sqrt{2\omega }}(1-i)$$

has to be added to circuit to fit the phase shift at low frequencies. This element represents semi-infinite linear diffusion and has only a small influence on the magnitude, proportional to the admittance (Y_W_). The most significant change we observed relates to the charge transfer resistance (R_ct_) after electrical stimulation: the parameter more than doubled, suggesting a much more capacitive behavior at the interface by reducing Faradic currents over the interface. This general trend can be observed in all the performed measurements (see supplementary information), although it does not seem to depend on the two carbon types. One way to interpret such behavior could be to assume that the so-called ‘active area’ of the electrodes decreases after the treatment. However, this would also reflect in the doubling of the electrodes’ real capacitance^35^ which – though – it is not observed in this study. When the Warburg impedance is neglected, according to Brug *et al*.^36^ the capacitance can be approximated to:

3 $$C={[T{(\frac{{R}_{ct}+{R}_{S}}{{R}_{ct}\cdot {R}_{s}})}^{n-1}]}^{1/n}$$

The determined values show little changes after the electrical and electrochemical treatments of the electrodes, excluding any significant contribution of the capacitance (and thus morphological changes) to their behavior. The EIS parameters extrapolated from the fitting are shown in Table 1.

**Figure 6:**
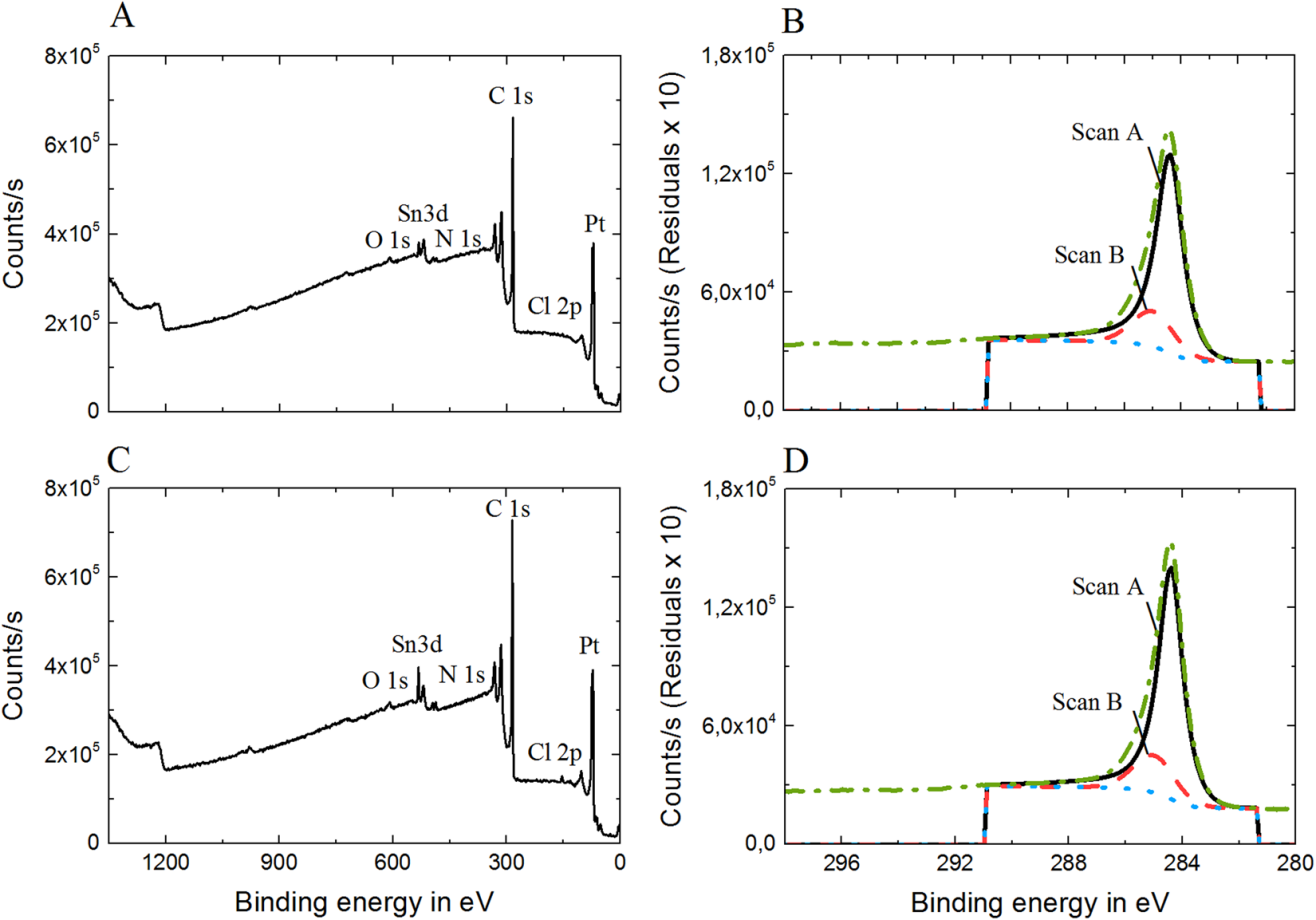
(**A**) Wide-scan or survey spectrum of a P20 laser-induced carbon electrode, showing all elements present; (**B**) XPS spectra of C 1s energy level for the same P20 sample. (**C**) Survey spectrum of a P40 laser-induced carbon electrode and (**D**) XPS spectra of C 1s energy level for the same P40 sample.

Elemental analysis of pristine, aged and electrically stimulated laser-induced carbon electrodes was performed for both groups through x-ray photoelectron spectroscopy (XPS). Survey spectra and the C 1s scans of a P20 and a P40 electrodes are shown in Fig. 6. The survey spectra of both samples (Fig. 6A,C) reveal a C 1s signal of high intensity in addition to other lower intensity peaks - belonging to products of the manufacturing process. The C 1s spectra are shown in Fig. 6B,D, where it is clear that the C 1s energy level is centered on 284 eV, typical binding energy (BE) value for graphite and hydrocarbon compounds^37^. The C 1s peaks of both carbon types are located in the same BE range and their intensity is comparable. In Table 2, the relative peak areas of different functional groups for each sample are examined. The analysis reveals an atomic percentage of graphitic carbon of 75% and 74%, for the pristine P20 and P40 electrodes respectively, and confirms that both laser parameters yield a graphitic type of carbon by pyrolyzing the parylene C insulation layer on top of the platinum-iridium tracks. These percentages are higher than the percentage of graphitic carbon present in polished glassy carbon electrodes found in literature (71.9%^31^, when the glassy carbon electrodes are thermally treated at 2500 °C, or 67.99%^37^). The percentage of graphitic carbon decreases (over 10%) for both the aged samples, and the same happens to stimulated electrodes belonging to the group P40, while the stimulated electrode in the group P20 remains similar to the pristine of the same group - with the exception of an increase in the oxygen-containing species (2–3%) and decrease in sodium-containing species (probably contaminants due to handling which randomly appear in some spectrograms). Additionally, the percentage of carbon bonded to other functional groups (C-H, here), as well as the ratio C_ox_/C_gr_ is calculated from the C 1s spectra of both groups and the determined values are listed in Table 2. These results indicate that the surface of the laser-induced carbon electrodes in the P40 group oxidizes during the aging and – especially – the biphasic pulse tests, while the P20 electrode type seems to be almost indifferent to the treatments. For the P40 type, in fact, the C_ox_/C_gr_ ratio increases both after the aging and stimulation tests.

**Table 2:** Relative quantities (%) of different compounds for various laser-induced carbon electrodes (given BE values are averaged values).

		Atomic %							
		C 1s^a)^	C 1s^b)^	O 1s^c)^	Na 1 s^d)^	P 2p3^e)^	Ir 4f7^f)^	Pt 4f7^g)^	C_ox_/Cgr
Pristine	P20	74.97	16.53	1.88	1.42	0.00	0.97	5.65	0.22
	P40	73.43	15.83	4.68	0.87	0.00	0.84	5.01	0.21
Aged	P20	62.48	9.73	9.19	6.07	1.31	0.40	2.73	0.15
	P40	57.63	15.16	6.86	8.54	1.18	1.43	7.89	0.26
Stimulated	P20	73.47	16.03	4.95	0.00	0.00	0.68	3.77	0.22
	P40	54.91	18.31	10.25	6.38	0.96	0.08	0.59	0.34

^a)^Graphite, 284.4 eV; ^b)^C-H, 285.0 eV; ^c)^Oxygen species, 532.2 eV; ^d)^Sodium species, 1072.9 eV; ^e)^PO_4_ -Phostphate species, 133.8 eV; ^f)^Ir, 60.5 eV; ^g)^Pt, 71.2 eV.

Nevertheless, the percentage of oxygen-containing species increases in all the cases, in comparison to the pristine ones; this phenomenon could possibly be ascribed to the presence of quinone and hydroquinone groups in the oxide layers forming and growing at the edge planes during the pre-activation phase of carbon electrodes, as already hypothesized by others^31,38,39^. It is known that in its reduced form, carbon mainly has hydroquinone-like groups, while in its oxidized form it has a pronounced quinonoid structure^40^. The transition from the reduced to the oxidized form starts when the carbonization temperature reaches 650 °C and the amount of quinonoid structures increases with the temperature increase: the higher the temperature reached during the carbonization process, the more oxidized the carbon and the more it is prone to activation^41^. It is also interesting to notice that the study conducted by Dekanski *et al*.^31^ has demonstrated that pyrolysis temperature has a high impact on the ability of a carbon electrode to get activated: pyrolyzed carbon electrodes thermally treated at 2500 °C are much more polarizable - and thus prone to activation - than the ones treated at 1000 °C. We can then assume that the temperature reached during our laser-carbonization processes is higher than 650 °C in both cases (P20 and P40), but P40 is most likely subjected to higher temperature during the manufacturing process and it is therefore more liable to be further activated. This corroborates the results obtained from the electrical stimulation of the P40-type electrodes (Fig. 4 and supplementary information), where we observe the decrease of the impedance magnitude and the increase of the charge storage capacity after biphasic pulse stimulation, both signs of activation^31^.

**Figure 7:**
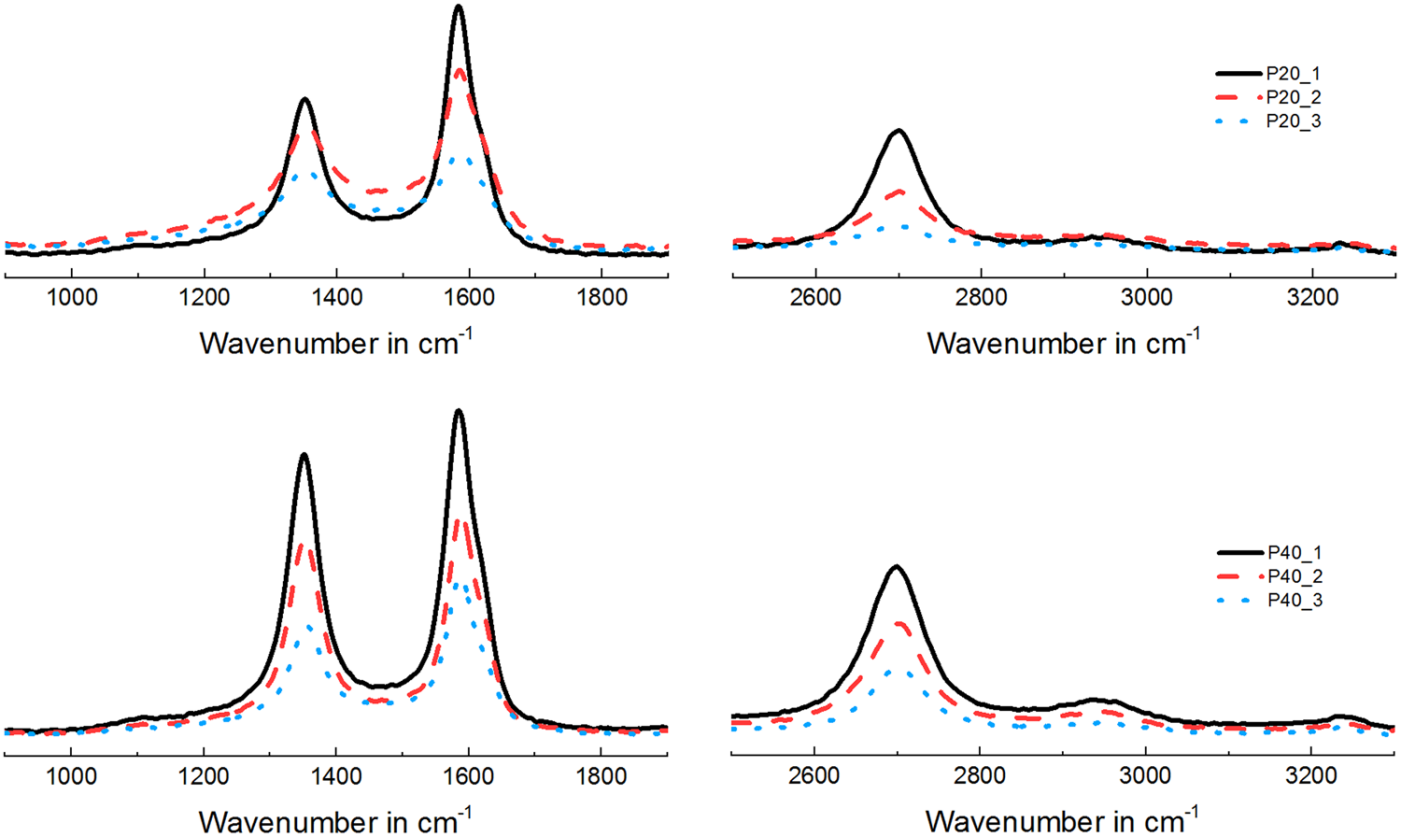
Raman spectroscopy of pristine electrodes P20 (top) and P40 (bottom). Three measurements were taken for each sample (in three random locations).

Raman spectroscopy was then performed to compare the level of crystallinity of P20 and P40. Results are shown in Fig. 7, where the Raman spectra of two pristine laser-induced carbon electrodes (P20 and P40-type) are divided into first-order (from 900 to 1900 cm^−1^) and second-order (from 2500 to 3300 cm^−1^) spectra. In the first-order zone, both the electrodes exhibit a peak at 1360 cm^−1^, called D band (as disorder), and one at 1575 cm^−1^, called the G band (after graphite). In non-organized (aka non-crystalline) structures, the first-order zone of the spectrum exhibits a very wide and asymmetric band with a maximum at 1600 cm^−1^. As a structure becomes more crystalline, the two bands (D and G) become distinguishable and with the further increase of the material’s structural order, the intensity of the D band reaches the same amplitude of the G peak (e.g. high modulus carbon fibers, similar to graphite but more ordered)^42^. To better quantify the level of crystallinity of the two carbon types, the relationship between D and G band intensity was calculated as the ratio I_D_/(I_D_ + I_G_), formula already used in the literature for accounting the percentage of disorder (or non-crystalline structural components)^42,43^.

The results, shown in Table 3, demonstrate that both carbon types exhibit a level of disorder - between 40 and 50% - comparable with the structural disorder found in surface-activated graphite^42^. In the second-order spectra, instead, it is possible to identify at least two other peaks: one at 2700 cm^−1^ - well pronounced and typically seen in well-organized structures - and another at 2950 cm^−1^ which has extremely low intensity, especially in the P20 sample, and which is typically found in activated-graphite spectra. This peak is generally used to distinguish activated graphite from conventional graphite. Both Raman spectra (first and second order) of the two carbon types (P20 and P40) indicate that the laser-induced carbon samples investigated are similar to surface-activated graphite, in terms of crystalline structure and level of disorder.

**Table 3:** Three P20 and P40-type samples with relative intensity of disorder calculated from their first-order spectra.

Sample	I_D_/(I_D_ + I_G_)/%			
	#1	#2	#3	Average
P20	42.2	46.5	39.7	42.8
P40	46.7	42.0	47.2	45.3

**Figure 8:**
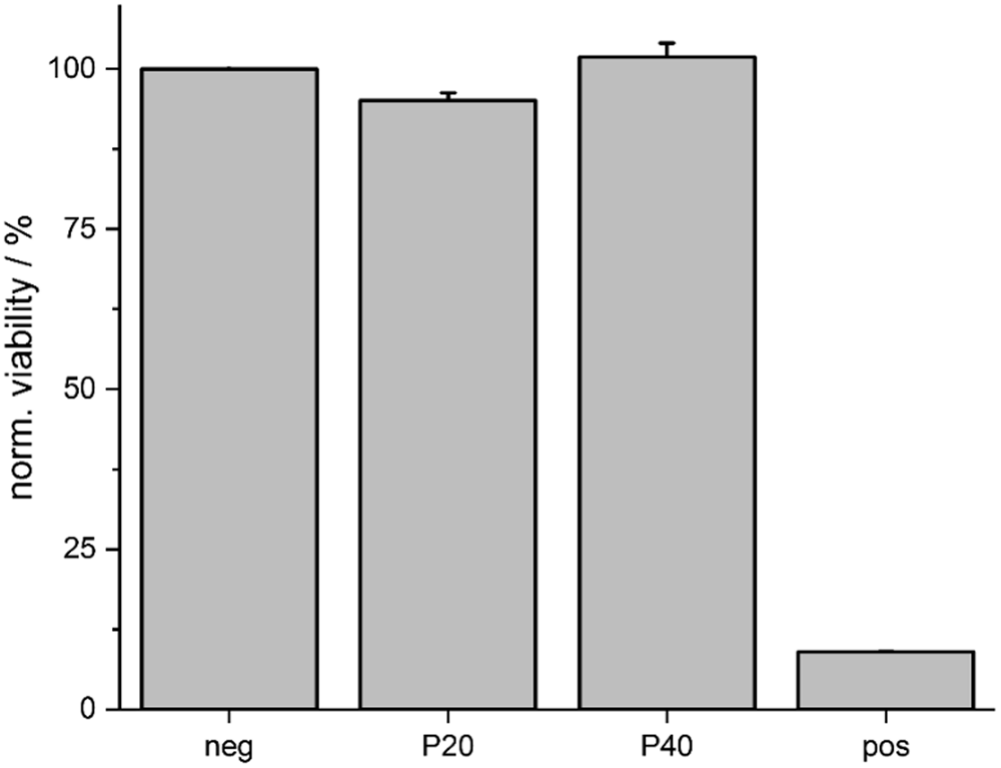
Quantitative analysis of the viability of neural cells cultured for 24 h in medium previously incubated with samples of laser-induced carbon P20 and P40 as well as normal cell medium (negative control). In the positive control, the cells were intentionally exposed to a toxic medium.

In order to study the biocompatibility of the laser-induced carbon electrodes, a cytotoxicity test was performed by using the elution method (see Experimental Section for details). For the test, three samples of each type (three for P20 and three for P40) were placed in a cell medium, which was subsequently used to grow human neuroblastoma cells. From each individual sample, as well as from the negative and positive controls, ten technical replicas were created and cells were counted to quantify their viability. Hereby, standard deviation was used to evaluate the weighted mean and derivation values for the two carbon types. Figure 8 represents the result of the test and shows that, for both carbon types, the percentage of surviving cells is comparable with the percentage of surviving cells of the negative control (i.e. non-toxic material). The positive control symbolizes the case where the majority of the cells die and the sample material is classified as cytotoxic. Both kinds of laser-induced carbon are considered non-cytotoxic according to the performed test, as their surfaces do not dissolve or release toxic residues in the cell culture medium.

One of the properties that differentiate carbon electrodes from other electrodes in neural applications is their ability to detect electrically active species, and thus neurotransmitters. For validating our technology and verifying the quality of the carbon yielded with our infrared nanosecond laser, the ability of laser-induced carbon electrodes to detect different concentrations of dopamine (DA) *in vitro* was investigated. Dopamine detection experiments were conducted using fast scan cyclic voltammetry (FSCV) as measurement technique and aimed to monitor the change in DA levels in phosphate buffered saline (PBS) solution by detecting current changes at the voltage (0.43 V, in this case) corresponding to the expected value of oxidation of the species of interest, in a setup previously used by others^44^. For this experiment, 200 µm-diameter pristine P20-type electrodes were utilized, as the high background current generated by larger electrodes (700 µm in diameter) prevented the identification of any eventual current change. Due to their structural and morphological affinities with the P20-type – a similar behavior is expected when the experiments were run using pristine P40-type electrodes.

**Figure 9:**
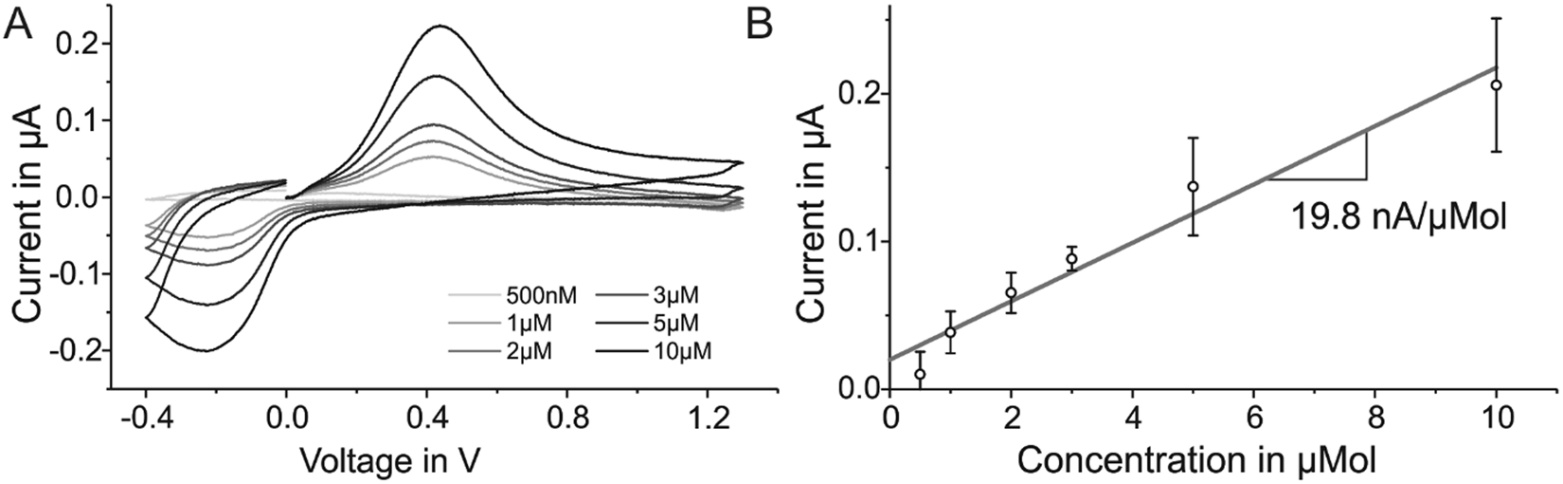
(**A**) Background-subtracted voltammograms of a representative P20-type carbon electrode when subjected to different dopamine concentrations (500 nM, 1 µM, 2 µM, 3 µM, 5 µM and 10 µM) in phosphate buffered saline solution and (**B**) calibration curve (grey line) obtained by plotting the average peak current (with standard deviation) over the concentrations of dopamine detected *in vitro* using three P20-type carbon electrodes (200 µm in diameter).

The electrodes were subjected to FSCV triangular waveforms between −0.4 V and +1.3 V with a resting potential of −0.4 V vs. a chlorinated silver wire reference electrode at 10 kHz repetition rate. The collected cyclic voltammograms were modified by subtracting the capacitive components of the measured current^45^ and show that the amplitude of the oxidation peak is proportional to the amount of DA present in solution (Fig. 9A). The background current generated by the tested P20-type pristine electrodes ranged between 0.8 and 2.0 µA at 0.43 V, voltage at which the oxidation peak of DA was observed. The substantial background current can be caused by the charging of the electrode double layer and redox reactions of electrochemically active surface groups^46^. An estimation of the electrodes performance was done by calculating their sensitivity index as the slope of the linear fitting of the averaged oxidation peak current measured per concentration of DA (Fig. 9B). The obtained value of ~19.8 nA/µM is in the range of values reported in literature for carbon fiber electrodes, accounting for the exposed geometrical surface area (GSA) of the active sites^47^. The limit of detection for the pristine P20-type was theoretically calculated to be 1.4 µM; however, it should be noted that this value could be greatly improved by tailoring the technology to the specific application with appropriate surface treatments and coatings.

## Conclusion

The intent of this study was to investigate a novel technology, completely based on the usage of a nanosecond laser, to determine whether it is suitable for short and long term neural applications. This technology allowed the rapid prototyping of carbon electrodes - by inducing with the laser the carbonization of parylene C - on top of robust metal sheets (e.g., platinum/iridium tracks) embedded in silicone rubber. The tracks and the device outlines were patterned by using the same laser. Two laser frequencies were used to make two types of carbon electrodes and to compare their properties and performance: 20 and 40 kHz. All the manufactured devices were aged in 30 mM H_2_O_2_ for one week, to mimic the post-surgery inflammatory reaction, and electrically stimulated - using biphasic pulses - up to 10 million times. The aim of these experiments was to test the robustness of the devices, and specifically of both kinds of laser-induced carbon electrodes and of the carbon-metal interfaces. The electrochemical behavior of the two carbon types in their pristine forms is very similar and does not seem to be significantly affected by the aging and stimulation. SEM images of the samples indicate that the electrodes did not delaminate, crack or detach from the metal substrate after the electrochemical treatment, nor during electrical stimulation. However, XPS analysis shows a higher percentage of oxygen-containing elements and higher C_ox_/C_gr_ ratio for the P40-type treated samples with respect to their pristine state. Raman spectroscopy puts our pristine electrodes in the disorder range of surface-activated graphite, proving that the laser carbonization process already initiates a certain degree of activation of the carbon surface. With such a comprehensive surface analysis, we were able to define the type of carbon obtained by lasering parylene C substrates with an infrared nanosecond laser. Laser-induced carbon electrodes were also proved to be non-cytotoxic and sensitive to dopamine concentration changes *in vitro*, even in the worst-case scenario (i.e. P20-type, untreated and non-activated). It must be also noted that the electrodes could undergo many cycles of fast scan cyclic voltammetry (400 V/s) during the dopamine detection experiments without failing, showing high stability under these parameters as well. This investigation proves that it is indeed possible to use an infrared nanosecond laser to both carbonize the electrode sites - and thus get the advantages of having carbon as interface with the brain tissue - and pattern the other components (devices outline and metal tracks). Both types of carbon pyrolyzed with the nanosecond laser are graphitic forms of carbon containing a high percentage of graphite (74% for the P20-type and 75% for the P40 type). The difference in laser pulse frequency likely reflects into a difference in temperature during the process: the P20 type of carbon, carbonized at 20 kHz, was subjected to lower temperature than the P40-type (carbonized at 40 kHz). Consequently, P40 was more likely to undergo electrochemical activation and oxidize. Moreover, both the carbon electrode types formed a stable compound with the underlying platinum iridium tracks (on top of which they are carbonized) and thus the well-known robustness of the laser-patterned devices was maintained. The comprehensive assessment of the laser-induced carbon electrode arrays *in vitro* indicates that they are non-cytotoxic and deliver adequate and stable electrochemical transfer characteristics for applications in neural recording, stimulation and neurotransmitter detection. The implantation of these probes and their assessment *in vivo* is the next step towards applications in neuroscientific research and clinical practice.

## Materials and Methods

### Device fabrication

The laser structuring of the devices used for this study was based on methods previously published and optimized^24–27^. Fabrication started with the lamination of a release layer (adhesive tape, No. 4124 by Tesa AG, Hamburg, Germany) on top of a ceramic carrier and the spin-coating of a 200 µm-thick layer of silicone rubber (PDMS, MED-1000, NuSil, Carpinteria, USA). Subsequently, a commercially available platinum-iridium (Pt-Ir) foil (with a thickness of 25 µm) was laminated onto it. The following steps consisted in structuring the metal foil with a pulsed nanosecond laser (DPL Genesis Marker Nd:YAG, ACI Laser GmbH, Deutschland), in removing the excess of the metal and in coating the entire device with a 10 µm layer of parylene C (DPXC by Specialty Coating Systems, Indianapolis, USA). Afterwards, the contact pads were opened and the outer line of the device was cut using the same laser. The last step, peculiar of this process, created the active sites of the array by laser pyrolysis of the insulation layer at desired locations (active sites) in a ‘line-writing’ manner. More precisely, in the specific case of the laser used for this study, this ‘writing’ technique consists in writing 10 µm-pitch lines to ‘fill in’ a certain geometry (in our case disk-shaped electrodes) and allows the carbonization of electrodes with a diameter of 200 µm or larger. Finally, the device was removed from the release layer. For this study, two types of carbon were produced: P20 and P40, which differ in the laser pulse frequencies used during the carbonization process (20 and 40 kHz, respectively). The laser power and the velocity of the moving beam used for both types of carbon are 1.6 W and 15 mm/s, respectively. A control group made of Pt-Ir electrodes was used to run control experiments. For making the control electrodes, the parylene C layer was selectively removed at the active sites instead of being carbonized. The final thickness of all the manufactured devices is 235 µm.

### Electrochemical characterization

All the manufactured electrodes were characterized electrochemically using cyclic voltammetry (CV) and electrochemical impedance spectroscopy (EIS) prior to the experiments. Both measurements were conducted in phosphate-buffered saline solution (PBS, pH = 7.4, Sigma Aldrich, USA), using a three electrode setup with a platinum counter electrode and Ag/AgCl (KCl 3 mol/L) reference electrode. Electrochemical interface was provided by a potentiostat and a frequency analyzer device (Solartron 1260&1287 by Solartron Analytical, Farnborough, Hampshire, UK) along with the software Zplot (v2.8 by Scribner Associates Inc., Southern Pines, NC, USA) to control the setup, and save and analyze the data. Initially, for each electrode, two sets of CV measurements were conducted (cleaning and characterization), followed by an EIS measurement. In the CV measurements, the vertex potentials were set between −0.8 and 0.6 V and the working electrode potential was first swept at a scan rate of 200 mV/s for 20 cycles and then at a scan rate of 50 mV/s for additional 50 cycles. The EIS measurements were made within the frequency range of 1 Hz to 10^5^ Hz with an excitation amplitude of 5 mV.

### Aging test

To evaluate the robustness of the electrodes during a potential implantation *in vivo*, the laser-induced carbon electrodes were aged in 30 mM hydrogen peroxide (H_2_O_2_) for one week at 37 °C. This test aims to simulate the acute post-surgery inflammatory reaction taking place in the animal’s brain tissue after an implantation. Therefore, four carbon electrodes per group (four P20-type and four P40-type electrodes) and one Pt-Ir control electrode were submerged in a PBS solution containing 30 mM H_2_O_2_ (H_2_O_2_ 30%, Carl Roth, Karlsruhe, Germany). Each electrode was placed in individual containers with a lid, containing 5 ml of the prepared solution. The containers were isolated from light with aluminum foil and covered with paraffin film to prevent evaporation. After been stored in an incubator (Heraeus, Germany) at 37 °C for one week, EIS measurements and CV were performed for all the electrodes.

### Biphasic current pulse stimulation

In order to study the stability of the laser-induced carbon under electrical stimulation, they were subjected to biphasic current pulses using an electrical stimulator (Plexon, Texas, USA). In total, 10 million pulses were applied to four electrodes of each laser-induced carbon group and to one control electrode (Pt-Ir). The experiment is conducted by applying trains of rectangular-shaped, symmetrical, biphasic, current-controlled, charge balanced and negative phase first waveforms. The selected pulse length of 300 µs and amplitude of 500 µA correspond to a charge density of 39.5 µC/cm² (one phase) applied at a repetition rate of 1 kHz. The adopted charge-per-phase corresponds to the median threshold used for retinal stimulation^48^. The measurements were conducted in PBS, using a two-electrode configuration setup, including the working electrode and a standard platinum counter electrode. EIS measurements were performed at intermediate stages for all electrodes and CV were performed after 10 million pulses.

### Surface characterization

The morphology of the electrodes in the pristine stage and after aging and stimulation was studied by scanning electron microscopy (Tescan Vega3 SEM - Tescan, USA).

### Elemental composition

The composition analysis of the laser-induced carbon electrodes was done using X-ray photoelectron spectroscopy (XPS). The measurements were performed using a K-Alpha+ XPS spectrometer (Thermo Fisher Scientific, East Grinstead, UK). For the data acquisition and processing was used a Thermo Avantage software, described elsewhere^49^. All samples were analyzed using a microfocused, monochromated Al Kα X-ray source (30–400 µm spot size). The spectra were fitted with one or more Voigt profiles (binding energy uncertainty: +/−0.2 eV). The analyzer transmission function, Scofield sensitivity factors^50^, and effective attenuation lengths (EALs) for photoelectrons were applied for quantification. EALs are calculated using the standard TPP-2M formalism^51^. All spectra were referenced to the C1s peak of hydrocarbon at 285.0 eV binding energy controlled by means of the well-known photoelectron peaks of metallic Cu, Ag, and Au. Sputter cleaning was performed using an Ar1000+ cluster ion beam at 8 keV and 30° angle of incidence which did not harm the electrodes. Raman spectroscopy was performed with the Raman microscope Bruker Senterra. The power of the laser was 5 mW with a wavelength of 532 nm. The integration time was 60 s with 2 co-additions (2 × 30 s) for each measurement spot.

### Cell viability

The cytotoxicity study was performed through an elution method. This method provided an extract of the target material which was then added to the culture cells. The cell line used for this study is the human neuroblastoma SH-SY5Y, which is commonly used for neural interfaces due to its neuron-like behavior. The cell culture medium consisted of 41 ml of Ham’s F12: EMEM (EBSS) (1:1), 2 mM Glutamine (Sigma D8062) with 7.5 ml of Fetal Bovine Serum (Sigma F0804), 500 µl Penicillin-Streptomycin (Sigma P4333) and 500 µl of MEM Non-essential Amino Acid solution (Sigma M7145). For each replicate, cells were cultured 24 h under sterile conditions in a humidified 5% CO_2_ atmosphere and at 37 °C in one well of a 96-well plate. In parallel, all samples were exposed to the test medium (cell medium without Fetal Bovine Serum) separately and in the same conditions. The medium of the cells was exchanged with the test medium exposed to the samples and incubated again for another 24 hours. After, 0,45 mg/ml Thiazol-blue-tetrazolin-bromide (Sigma M5655) was added to each well and the plate was incubated for another 2 hours. Medium was removed and the converted dye suspended in 50 µl DMSO. Afterwards, viability was quantified by measuring absorbance at 540 nm using a plate reader (Perkin Elmer, USA). Cultured cells in ordinary test medium were taken as negative control and cultured cells where all cells were intentionally killed with Triton-X 100 (1:10) were taken as positive control.

### Dopamine detection

Fast scan cyclic voltammetry (FSCV) was performed in a beaker where an invilog-setup (Invilog Ltd., Finland) was used to generate the waveforms and to collect the resulting FSCV data. A chlorinated silver wire (0.5 mm in diameter, Goodfellow) was used to serve as the reference electrode. The P20-type carbon electrodes were preconditioned utilizing a triangular waveform held at 0 V and changing the potential from −0.4 V to 1.3 V, with a scan rate of 400 V/s. Prior to each calibration, a baseline measurement was performed to let the electrode reach a steady response^45^. To accelerate the equilibrium process, a waveform in higher repetition rates (60 kHz) than the data collection frequency was used (10 min duration). Following the preconditioning step, all samples were again cycled for a period of 20 min at 10 kHz to initiate the baseline data set for the calibration^44^. The background subtraction was done by subtracting the recorded FSCV from an averaged CV set (10 cycles in PBS only) prior to each first injection^52^. A stock solution of 10 mM dopamine-hydrochloride (Sigma-Aldrich, St. Louis, MO) in phosphate buffered saline solution was freshly prepared for each experiment. Measurements were conducted for different concentrations of dopamine, i.e. 500 nM, 1 µM, 2 µM, 3 µM, 5 µM and 10 µM, eventually added to the stock solution^53^.

### Adhesion test

The adhesion between the laser-induced carbon and the metal layer was tested following a standard Scotch Tape test for measuring adhesion of structures to a substrate (D3359-07 from ASTM International, United States). The procedure consisted in producing an array of 10 × 10 laser-induced carbon sites with a diameter of 700 µm, 1 mm apart from each other. The tape was applied and pressed, to ensure a good contact with the layer underneath. After approximately 90 seconds, the tape was removed back upon itself with an angle of 180°. This procedure was performed according to the standard procedure^54^.

## Electronic supplementary material

Supplementary Information
