# Supplementary material for: Graphitic Carbon Electrodes on Flexible Substrate for Neural Applications Entirely Fabricated Using Infrared Nanosecond Laser Technology
Source: Sci Rep. 2018 Oct 3;8:14749. doi: 10.1038/s41598-018-33083-w (PMC6170440; doi:10.1038/s41598-018-33083-w)
Supplement: Supplementary file 1 — Supplementary Information [file 41598_2018_33083_MOESM1_ESM.docx]

Graphitic Carbon Electrodes on Flexible Substrate for Neural Applications Entirely Fabricated Using Infrared Nanosecond Laser Technology

Maria Vomero‡^1,2*^, Ana Oliveira‡^1^, Danesh Ashouri^1,2^, Max Eickenscheidt^1*^, Thomas Stieglitz^1,2,3^

^1^ Laboratory for Biomedical Microtechnology, Institute of Microsystem Technology (IMTEK), University of Freiburg, Georges-Koehler-Allee 102 D-79110 Freiburg, Germany

^2^ Cluster of Excellence BrainLinks-BrainTools, University of Freiburg, Georges-Koehler-Allee 80, 79110 Freiburg, Germany

^3^ Bernstein Center Freiburg, University of Freiburg, Hansastrasse 9a, 79104 Freiburg

‡These authors contributed equally

*address correspondences to [maria.vomero@imtek.de](mailto:maria.vomero@imtek.de) and max.eickenscheidt@imtek.de

**KEYWORDS** graphitic carbon; infrared laser technology; neural interfaces; soft electronics; neural recording; neural stimulation; electrochemical stability; microelectrodes.

**Supplementary Information**


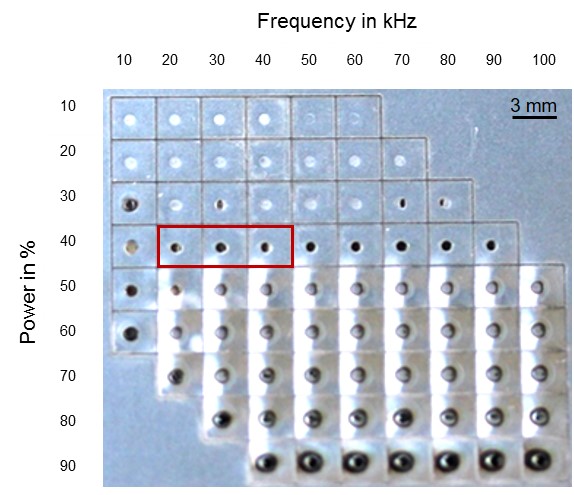


**Figure S1**. Array of laser-induced carbon electrodes from parylene C using various combinations of power (10 to 90 %, from top to bottom) and frequency (10 to 100 kHz, from left to right). Each circular electrode measures 700 µm in diameter. Range of combinations considered viable are circled in red in the picture. Other combinations either led to burned polymers (lower right) or little or no carbonization (upper left), respectively.

**
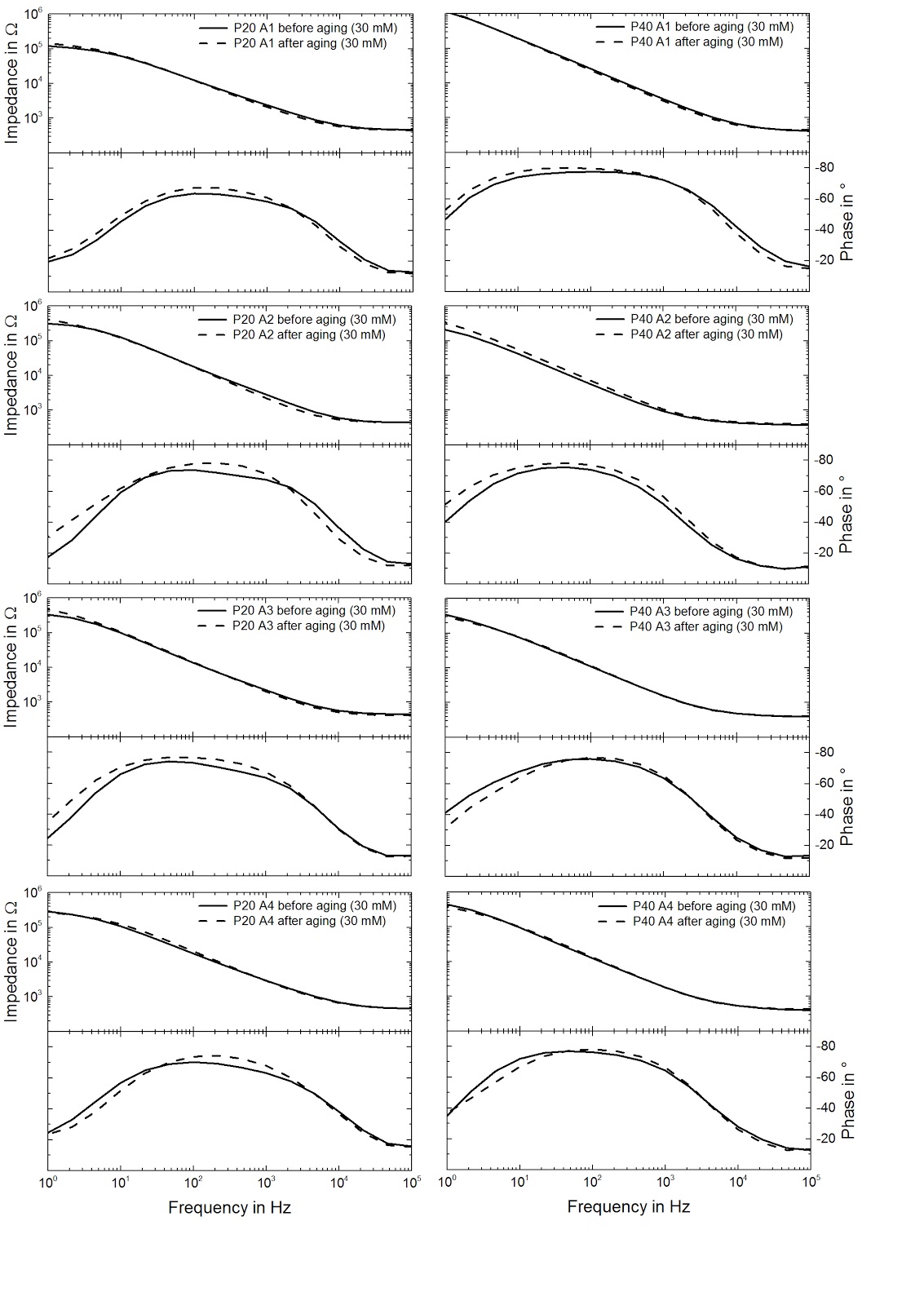
Figure S2**. EIS measurements (magnitude and phase) of P20 (left column) and P40-type (right column) carbon electrodes before and after aging in 30 mM H_2_O_2_ for one week at 37 °C.


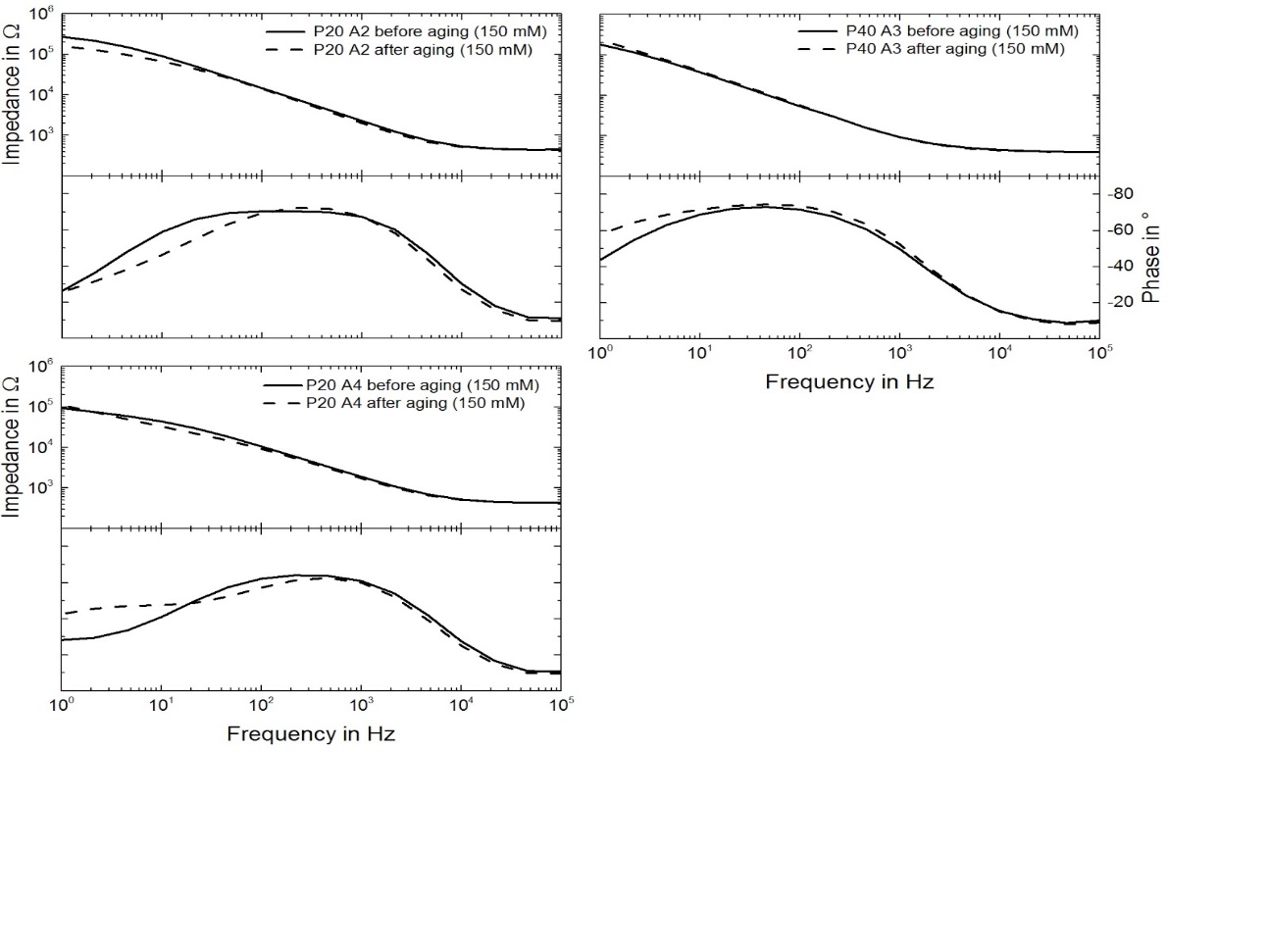
**Figure S3**. EIS measurements (magnitude and phase) of electrode P20 (left column) and P40-type carbon electrodes (right column) before and after aging in 150 mM H_2_O_2_ for one week at 37 °C.

**
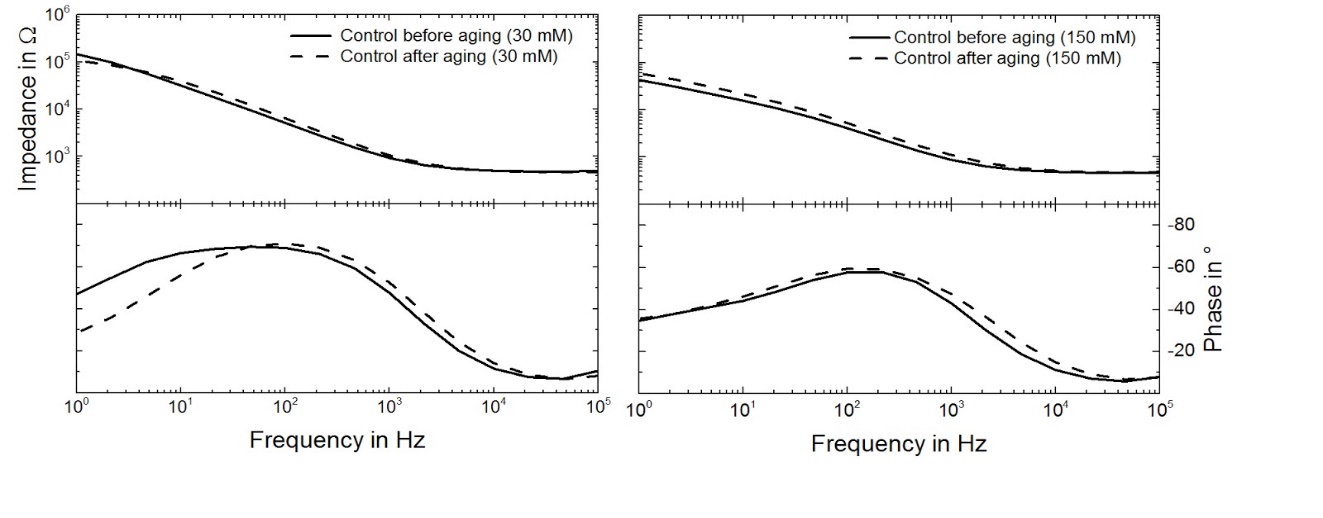
Figure S4**. EIS measurements (magnitude and phase) of the platinum-iridium control electrode before and after aging in 30 mM H_2_O_2_ and in 150 mM H_2_O_2_ for one week at 37 °C.

**
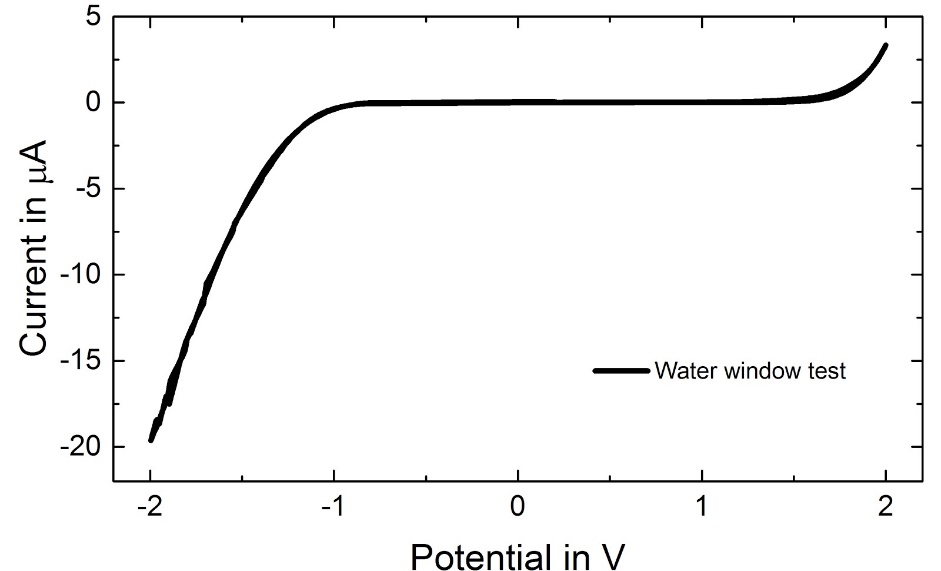
**

**Figure S5**. Cyclic voltammogram of a P40-type carbon electrode, cycled between -2 and 2 V; peaks of current occur at approximately -1 and 1.7 V and indicate electrolysis, i.e. the limits of the water window.


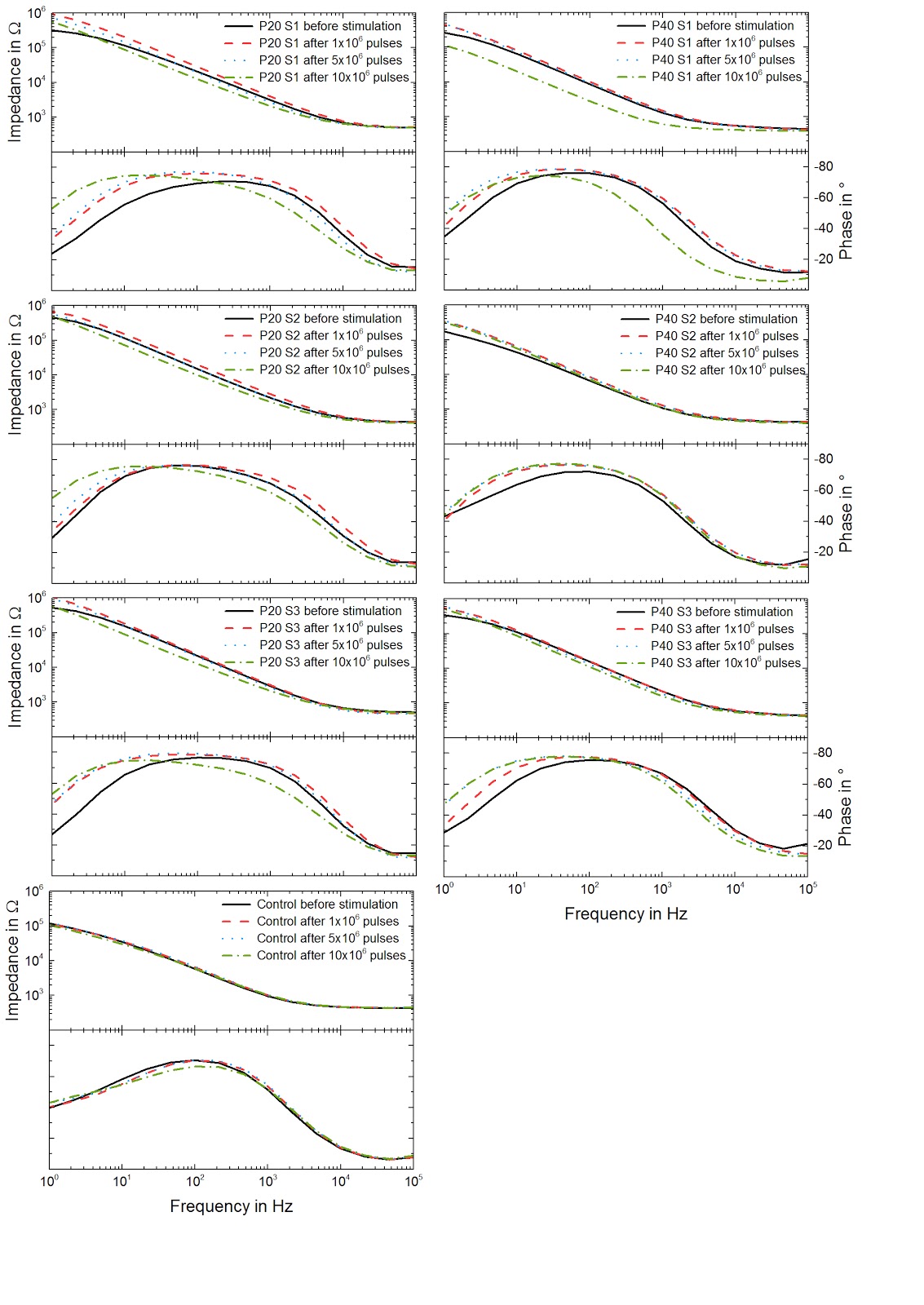
**Figure S6.** EIS measurements (magnitude and phase) of P20 (left column), P40-type carbon electrodes and control (bottom left column) before and after 1, 5 and 10 million biphasic current pulses.


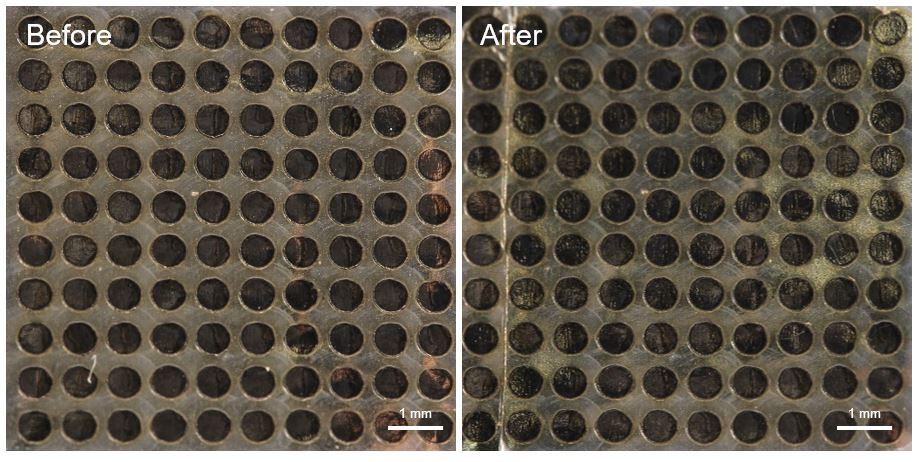


**Figure S7.** Array of laser-induced carbon sites before (left) and after (right) the Scotch Tape test. The pictures show no significant difference between electrodes before and after the Scotch Tape test, as none of them was damaged or detached from the substrate. This indicates that laser-induced carbon electrodes strongly adhere to the metal underneath.


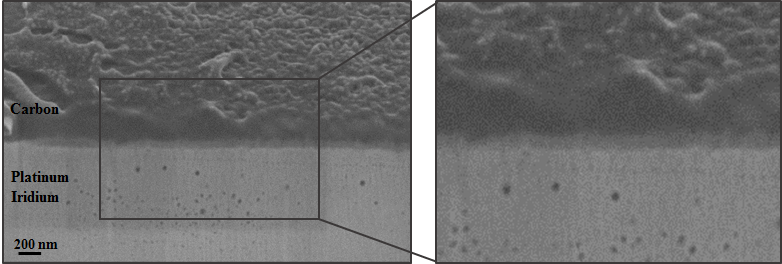


**Figure S8**. Focused ion beam (FIB) picture of a laser-induced carbon electrodes on the platinum iridium track. The carbon adheres well to the underlying metal.

**Table S1**. Percentage of graphitic carbon, averaged charge storage capacity (CSC) and impedance magnitude at 1kHz of pristine, aged and electrically stimulated carbon electrodes (n=4).

|  | Pristine | | After aging | | After stimulation | |
| --- | --- | --- | --- | --- | --- | --- |
|  | P20 | P40 | P20 | P40 | P20 | P40 |
| C-graphite / Atomic % | 75 | 74 | 62 | 56 | 73 | 55 |
| CSC / µC/cm^2^ | 54 | 34 | 98 | 43 | 30 | 42 |
| Impedance at 1 kHz /kΩ | 2.6 | 1.8 | 2.3 | 1.9 | 1.8 | 1.3 |
